# Supplementary material for: Effect of Lipids in Yak Muscle under Different Feeding Systems on Meat Quality Based on Untargeted Lipidomics
Source: Animals (Basel). 2022 Oct 18;12(20):2814. doi: 10.3390/ani12202814 (PMC9597711; doi:10.3390/ani12202814)
Supplement: Supplementary file 1 [file animals-12-02814-s001.zip › Table S1.pdf]

**Table S1** The common nutrition and content of main fatty acid in grass, TMR (air-dry basis).

| Item | TMR | Natural grass |
|---|---|---|
| Common nutrition | | |
| Crud fat | 4.52 | 2.63 |
| Crud peotein | 16.96 | 11.93 |
| Neutral detergent fiber | 23.24 | 76.14 |
| Acid detergent fiber | 13.84 | 10.09 |
| Calcium | 0.79 | 5.22 |
| Phosphorus | 0.37 | 0.07 |
| Fatty acid | | |
| C16:0 | 0.68 | 0.30 |
| C18:0 | 0.21 | 0.09 |
| C18:1 | 0.18 | 0.07 |
| C18:2n6 | 0.58 | 0.29 |
| C18:3n3 | 1.42 | 0.68 |
